# Evaluation of the Level of Dural Sac Tip in Saudi Population: A Magnetic Resonance Imaging Study

**DOI:** 10.7759/cureus.32533

**Published:** 2022-12-14

**Authors:** Mohammed S AL Jawad, Mazen A Alhomoud, Fadel M Alfaraj, Firas N Almuhaimeed, Fahad A AlDawsari, Saeed A AL-Jubran, Faisel A Albalawi, Syed Rehan H Daimi

**Affiliations:** 1 Radiology, College of Medicine, Imam Abdulrahman Bin Faisal University, Dammam, SAU; 2 Radiology, King Fahad University Hospital, Alkhobar, SAU; 3 Anatomy, College of Medicine, Imam Abdulrahman Bin Faisal University, Dammam, SAU

**Keywords:** craniospinal irradiation (csi), caudal block, vertebrae, spine, saudi population, mri, dural sac tip

## Abstract

Background

For the success of procedures such as caudal block, craniospinal irradiation (CSI), and management of lower back pain and to minimize the risk of dural puncture the exact level of dural sac (DS) termination should be known.

Objective

The evaluation of DS tip location in the Saudi population and exploring possible significant factors that could be used as predictors in clinical prognosis.

Methods

A total of 200 patients’ lumbar sagittal Weighted T2 Magnetic Resonance Imaging (MRI) study were randomly selected from a single-center hospital in-between 2020 and 2021. The DS tip location was determined by generating a perpendicular line from the longitudinal axis of its termination to the corresponding level. Then naming it after an intervertebral disk or a corresponding vertebrate that is divided into three thirds (upper, middle, and lower).

Results

In most cases, the level of DS termination is at the middle part of S2 (26.5%), followed by the upper part of S2 (25.1%), and the lower part of S2 (20%). In Saudi nationals, the DS tip was in the middle S2 level at 21.5%, upper S2 level at 19.1%, and lower S2 level at 17%. Factors such as age, sex, cause of referral, and nationality had no statistical significance in relation to DS tip location.

Conclusion

The DS termination level in the Saudi population ranges from disk between L5-S1 to the lower third of S3. Moreover, nationality, age, and cause of referral were not significant in determining the DS termination level. Therefore, it is still important to individualize patients’ treatment by using MRI for each case that requires it.

## Introduction

The spinal meninges are composed of three layers. The innermost layer is called the pia mater. The middle layer is called the arachnoid mater. The outermost layer is called the dura mater. The dural sac (DS), also known as the thecal sac, is composed of the dura and arachnoid mater. It contains cerebrospinal fluid (CSF) and spinal nerves. In classic textbooks, the DS termination is considered at S2, whereas in real life it is variable and other factors could potentially affect the level of termination as well [1-3].

Various methods were used to identify the DS termination level in the past. Practitioners were previously relying on cadaveric assessment. However, it was discovered to be limited clinical benefit [4,5]. Other forms of practice included an invasive procedure known as myelography. It was performed by injecting contrast into the spine in combination with imaging studies such as X-rays and computed tomography (CT) scans [6,7]. Nowadays, myelography is replaced by magnetic resonance imaging (MRI) which is widely known for its effectiveness in locating the termination level of DS and in demonstrating the anatomy of the lumbosacral region. It can effectively take images of the spine in multiple planes which aids in the identification of possible spinal disorders. Additionally, it does not require an injection of contrast material making the procedure more appealing to patients’ preferences, increasing the level of acceptance in terms of needle phobia and anxiety toward pain, and it doesn’t pose any biological risks [8,9]. In light of this, MRI was chosen to identify the levels of DS termination in living patients using sagittal T2-weighted images of the lumbosacral area.

The termination level of DS is significant to anesthesiologists in a number of settings, such as when performing caudal epidural blocks (CEB), as it can potentially reduce the rate of complications [10]. Orthopedic surgeons can benefit from acknowledging the DS location for performing epidural steroid injections to relieve chronic leg and back pain resulting from various conditions such as herniated discs [11,12]. Lumbar puncture is a diagnostic and potentially therapeutic procedure for some disorders which could benefit from the DS termination level [13]. Radiotherapists and radiation oncologists benefit from this point as well. Neglecting DS termination level while performing craniospinal irradiation (CSI) can lead to failure of the procedure [7].

For the success of various surgical and diagnostic procedures in the lumbosacral region and to minimize the risk of puncturing and failure, it is essential to have knowledge of the termination level of DS to the corresponding vertebra. Variations in the termination of DS have been described in various population groups.

In Saudi Arabia, the number of studies regarding DS termination is inadequate and insufficient. This allows more opportunities to conduct more studies on this topic to explore the potential of using the knowledge of DS tip in clinical practice to the extent.

## Materials and methods

Study design, setting, and duration

A cross-sectional study was conducted on patients who had undergone a mid-line sagittal T2 weighted MRI showing the lower back at King Fahad University Hospital, Alkhobar, Saudi Arabia. A total of 200 patients fulfilling the study criteria were randomly selected from August 2020 to November 2021. 

Inclusion criteria

The inclusion of patients was based on MRI availability, age (between 18 to 70), and absence of any pathology or developmental defects that could affect the DS tip location.

Data collection

The study was approved by the Institutional Review Board (IRB) of Imam Abdulrahman Bin Faisal University (IAU), and permission was obtained from Radiology Department in King Fahad University Hospital, Alkhobar, Saudi Arabia. Data were extracted from the electronic record system to a standardized Microsoft Excel spreadsheet (Microsoft, Redmond, Washington). The sheet includes the date of birth, sex, nationality, and cause of referral.

Image analysis

An MRI of the spine was conducted using a 1.5 Tesla MRI machine (Siemens, Munich, Germany) while the patient lay supine. Sagittal images were acquired using a slice thickness of 3.0 millimeters (mm). In spite of both T1- and T2-weighted sagittal images being available, T2 sagittal images were better at illustrating the relevant anatomy. Furthermore, many studies of this kind favored T2-weighted imaging. Thus it provided conformity to our methodology, allowing further comparability between results. 

**Figure 1:**
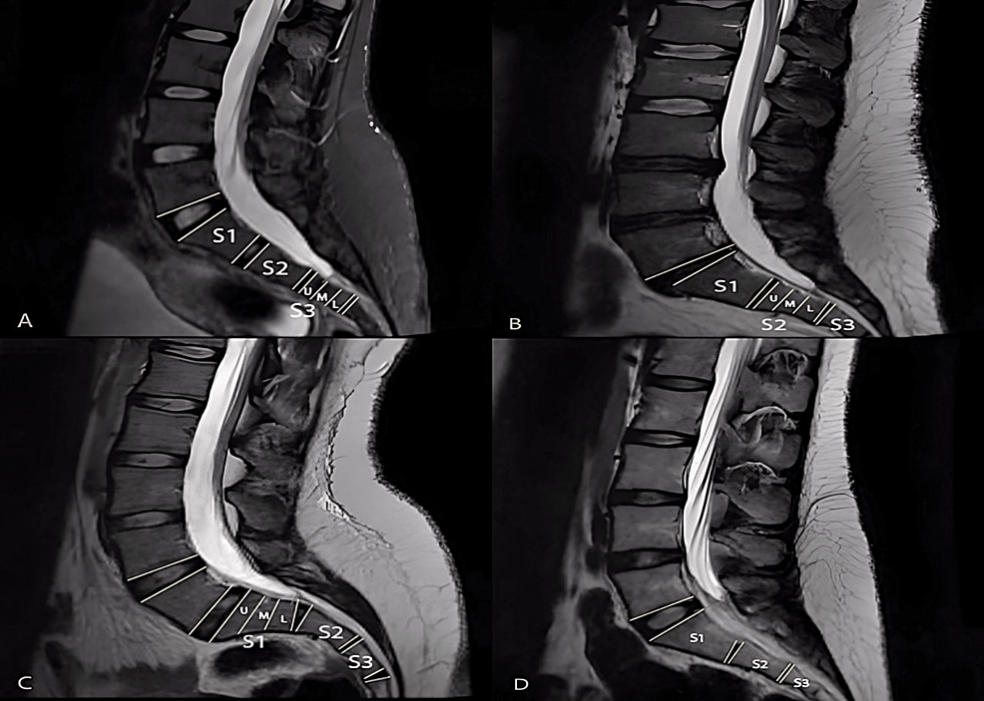
T2 weighted MRI images with various DS termination levels (A) The DS Tip at the S3 Upper level. (B) The DS Tip at the S2-Middle level. (C) The DS Tip at the S1-Lower level. (D) The DS Tip is at the space L5-S1 Disk level MRI: Magnetic resonance imaging; DS: Dural sac

The DS tip location was determined in relation to the corresponding vertebrae level by one of the authors in the radiology department. The corresponding vertebrae or vertebral space is defined using Saifuddin et al.'s method [14], by generating a perpendicular line from the DS Tip longitudinal axis to the corresponding level. Then, the corresponding vertebrate is divided into three parts (upper, middle, lower); however, the intervertebral spaces are considered as one segment and were named by their prior and subsequent vertebrae (Figure 1).

Statistical analysis

Data were included in a process of coding. Afterward, the continuous variable (age) was described as mean ± standard deviation, and categorical variables were described as frequencies and percentages. Comparative analyses to assess patterns of DS tip locations were assessed via a set of T-tests for the continuous variable and Chi-squared tests. Fisher’s exact test was used whenever the expected count was less than 5. P-value was set to 0.05. All analyses were performed in SPSS statistical software version 15.0 (SPSS Inc., Chicago, IL).

## Results

The total number of sampled patients was 200. The mean age of all patients was 41.91 ± 10.92 years. Females made up 60% of the entire sample and 84% were non-Saudis. Frequency distribution for the level of DS termination was mostly located in S2 (71.50%). Examining these in more detail, 26.50% were in the middle third, 25% in the upper third, and 20% in the lower third of S2. The main reason for referral was back pain among this sample of patients. Non-specified back pain accounted for 64.70% of all patients, while only three patients were referred due to numbness (Table 1). 

**Table 1:** Patient demographic and clinical characteristics

Characteristics	N (%) 200 (100.00)
Age (X̄, σ_x_)	41.91 (10.92)
Gender	
Males	80 (40.00)
Females	120 (60.00)
Nationality	
Saudi	168 (84.00)
Non-Saudi	32 (16.00)
Dural Sac Tip Location	
S1-Lower Third	24 (12.00)
S1-Middle Third	3 (01.50)
S2-Lower Third	40 (20.00)
S2-Middle Third	53 (26.50)
S2-Upper Third	50 (25.00)
S3-Lower Third	2 (01.00)
S3-Middle Third	5 (02.50)
S3-Upper Third	14 (07.00)
L5-S1	1 (00.50)
S1-S2	6 (03.00)
S2-S3	2 (01.00)
Cause of referral	
Non-specified back pain	121 (64.70)
Chronic back pain	14 (07.50)
Lower back pain	37 (19.80)
Severe back pain	12 (06.40)
Numbness	3 (01.60)

**Figure 2:**
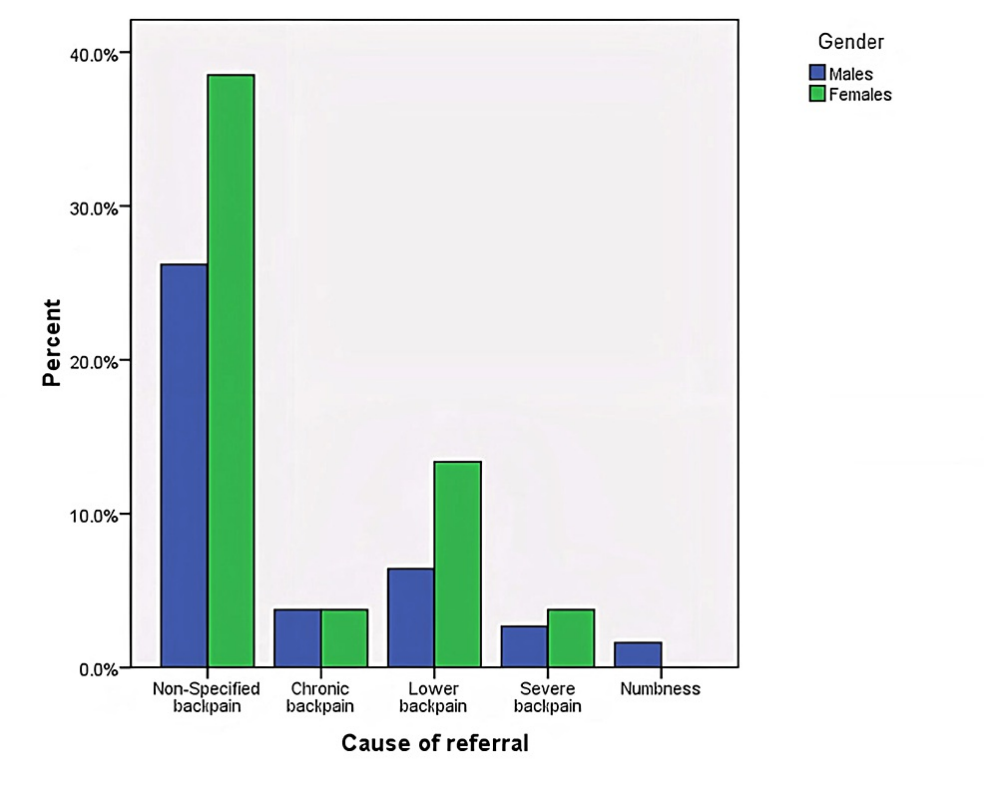
The cause of referral of patients according to their gender

Figure 2 shows the cause of referral according to the patient’s gender. The majority of patients who complained of back pain were females.

Table 2 presents the pattern of DS termination by the demographic characteristics of patients (where the number of patients exceeded 5 for each location). Although no significant differences were observed for any of those locations, the mean age of patients was highest for patients with a DS termination location at the upper third of S3, followed by the lower third of S2 (mean age = 46.93 ± 12.48 and 43.78 ± 09.74 years respectively). Patients with a DS tip location in the S1-S2 spaces were relatively younger (mean age = 33.67 ± 9.91 years).

**Table 2:** DS tip location by demographic characteristics of all sampled patients ‡Locations with frequencies exceeding five patients only.

Characteristics	DS Tip location^‡^						P-value
	S1-L	S2-L	S2-M	S2-U	S3-U	Space S1-S2	
Age (X̄, σ_x_)	38.17 (10.87)	43.78 (09.74)	42.11 (11.21)	41.86 (11.38)	46.93 (12.48)	33.67 (09.91)	0.07
Gender							
Males	6 (07.90)	15 (19.70)	26 (34.20)	21 (27.60)	4 (05.30)	4 (05.30)	0.24
Females	18 (16.20)	25 (22.50)	27 (24.30)	29 (26.10)	10 (09.00)	2 (01.80)	
Nationality							
Saudi	22 (14.20)	34 (21.90)	43 (27.70)	38 (24.50)	13 (08.40)	5 (03.20)	0.52
Non-Saudi	2 (06.20)	6 (18.80)	10 (31.20)	12 (37.50)	1 (03.10)	1 (03.10)	
Cause of referral							0.62
Non-specified backpain	17 (14.00)	27 (22.30)	37 (30.60)	30 (24.80)	7 (05.80)	3 (02.50)	
Chronic backpain	3 (21.40)	2 (14.30)	3 (21.40)	5 (35.70)	1 (07.10)	0	
Lower backpain	3 (08.10)	7 (18.90)	10 (27.00)	13 (35.10)	2 (05.40)	2 (05.40)	
Severe backpain	1 (08.30)	3 (25.00)	2 (16.70)	2 (16.70)	3 (25.00)	1 (08.30)	
Numbness	0	1 (33.30)	1 (33.30)	0	1 (33.30)	0	

As far as gender is concerned, the most common DS tip location for males was the middle third of S2 (34.20%). By contrast, the most common location for DS tip in females was in the upper third of S2. As far as nationality is concerned, the most common location for DS tip in Saudis was in the middle third of S2 compared to non-Saudis (27.70% and 37.50% respectively).

Among patients with non-specified back pain, 30.60% had a DS termination location at the middle third of S2. On the other hand, chronic back pain and lower back pain groups of patients (35.70% and 35.10% respectively), had a DS termination location in the upper third of S2. Patients with severe back pain had the DS tip location to be terminated at the lower third of S2 and the upper third of S3 but had an equal percentage of 25%.

**Figure 3:**
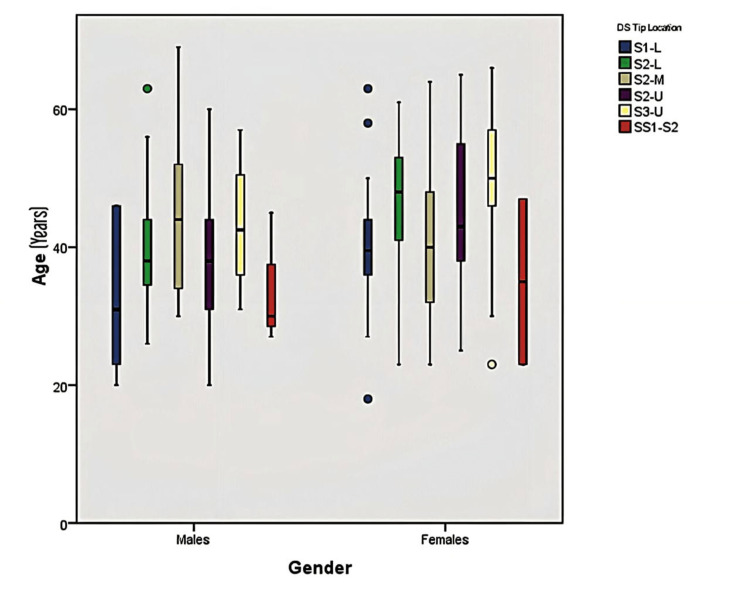
Distribution of age among males and females according to DS tip location L: Lower, M: Middle, U: Upper, S: Space

Figure 3 gives the pattern of the different DS tip locations by age and gender. For the lower third of S1, the age of males ranged between 20 and 46 years, whereas females' age ranged between 26 and 50 years. Also, the median age of females in patients within the lower third of S2 was higher than that among males. Differences also existed in the age range and median age between males and females in both the upper third of S2 and the space between S1 and S2.

## Discussion

Several studies have determined the termination level of DS in adults by conducting cadaveric and MRI-based studies in different populations [4,5,8,15]. We conducted a cross-sectional MRI-based study in the previously unstudied Saudi population to observe and document the termination level of the DS.

Our study showed a spectrum of DS termination that ranged from the L5-S1 disc space to the lower third of S3 which is consistent with the previous studies [15,16]. However, other studies demonstrated that DS terminates from the S1 to S4 [8,17]. Trinh et al. found that DS terminates from the upper border of S1 to the lower border of S3 [18]. Based on the result of this study, we found that DS most commonly terminates at the middle third of S2, which is in accordance with a study done in South Africa by Cilliers et al. [16]. In contrast, MRI studies in Turkey that measured the termination of the DS showed a mean level at the upper third of S2 [15,17]. Additionally, a British study determined that S2 terminates at the upper third of S2 as well [8]. Binokay et al. hypothesized that these differences in the termination levels of DS might be explained by racial differences and techniques applied among each study sample [15]. The results of this study concur with most studies in the literature showing no statistical significance between DS termination and age [15-17,19-21]. Although, one study found that age had a small but systematic effect on the position of the DS and age [22]. As in other studies, we found that male and female patients showed no statistically significant differences in DS position [4,15,20,21]. In contrast to Macdonald et al. findings, which showed an association between age and DS location [8]. According to our findings, there is no statistical difference between DS termination levels and race or nationality and this is consistent with Cilliers et al. findings that could not detect a difference between races of blacks and whites in South African populations [16].

Defining the termination level of DS is crucial in many clinical applications like CSI, which is one of the most commonly practiced radiation techniques for treating patients with intracranial tumors [21]. In order to achieve consistent results with this procedure, it is crucial to include the distal DS in the CSI spinal field to completely cover the central nervous system [21].

CEB is a procedure administered for the purpose of managing chronic back pain in both adults and children [23]. Despite having competent clinical skills and experience in the hands of a healthcare practitioner, the failure rate of this procedure remains high especially in adults and while using a blind technique [23]. The high failure rate can also be attributed to the anatomical variation of the DS level of termination [23,24]. This procedure is done by inserting a needle in the sacral hiatus and accessing the epidural space [23]. Acknowledging the significance of anatomical variation by a healthcare provider can be crucial in terms of avoiding complications in this procedure like puncturing the dura [23,25-27]. In addition, healthcare providers responsible for practicing this procedure clinically should consider the difference in the anatomical variation of the sacral hiatus as well as the distance between DS and sacrococcygeal membrane in the patients under treatment [23,28,29]. While fluoroscopy and ultrasonography can help in guiding the healthcare provider and reducing the risk of complications, it is highly advisable for the physician to perform an MRI prior to conducting this procedure for the patient to detect the presence of any anatomical variation, Tarlov cysts, lumbosacral transitional vertebrae and to ensure adequate visualization of the entire spinal anatomy [10,20,21,23]. Patients with Tarlov cysts are at higher risk of their dura being punctured than the normal population [25].

## Conclusions

The present study aimed to explore the variations of DS tip location among the Saudi population. This study discovered that most levels of DS termination were in the middle part of S2 (26.5%), followed by the upper part of S2 (25.1%), and the lower part of S2 (20%). There were no cases found of the DS tip being in L5 or upper S1. Also, this study found an association between corresponding vertebral length and DS tip location. And the mean score of females was lower than males after measurement by the independent t-test. However, age and sex had no statistical significance in relation to DS tip location. Hence, it is important for clinicians to understand the potential differences in the location of the DS termination while performing a lumbar puncture for any indication. As evidence remains insufficient in this topic, there is no significant indicator of DS tip location without imaging; therefore, it is advisable to perform MRI prior to any surgical intervention in this region. Further research on this topic is highly recommended to utilize DS tip location for therapeutic procedures to reach maximum benefit and to explore this field and comprehend its potential in clinical practice to the extent.
