# Correction: Evaluation of the Level of Dural Sac Tip in Saudi Population: A Magnetic Resonance Imaging Study

**DOI:** 10.7759/cureus.c94

**Published:** 2023-01-12

**Authors:** Mohammed S AL Jawad, Mazen A Alhomoud, Fadel M Alfaraj, Firas N Almuhaimeed, Fahad A AlDawsari, Saeed A AL-Jubran, Faisel A Albalawi, Syed Rehan H Daimi

**Affiliations:** 1 Radiology, College of Medicine, Imam Abdulrahman Bin Faisal University, Dammam, SAU; 2 Radiology, King Fahad University Hospital, Alkhobar, SAU; 3 Anatomy, College of Medicine, Imam Abdulrahman Bin Faisal University, Dammam, SAU

This article has been corrrected at the request of the authors to include Dr. Syed Rehan H. Daimi as last author. Dr. Daimi was erroneously omitted from the author list by the submitting author and instead included in the Acknowledgements section of the article. Upon realizing their error, the authors contacted Cureus to request that Dr. Daimi be added. Dr. Daimi also contacted the journal with this request. Upon reaching a consensus, Dr. Daimi was added as last author, the corresponding Acknowledgement removed, and the formal correction issued. The authors deeply regret this error and apologize to Dr. Daimi for the omission.

